# Comparative mitogenomics of marine angelfishes (F: Pomacanthidae)

**DOI:** 10.1002/ece3.70127

**Published:** 2024-08-08

**Authors:** Lauriane M. Baraf, Julia Y. Hung, Morgan S. Pratchett, Peter F. Cowman

**Affiliations:** ^1^ College of Science and Engineering James Cook University Townsville Queensland Australia; ^2^ Biodiversity and Geosciences Program Queensland Museum Tropics Townsville Queensland Australia

**Keywords:** bioinformatics, mitogenome, off‐target reads, Pomacanthidae, reef fish, ultraconserved elements

## Abstract

The targeted capture of ultraconserved elements (UCEs) has substantially increased the amount of genetic data available for phylogenomic reconstructions. These capture datasets frequently contain mitochondrial DNA as a by‐product, often in the form of complete mitogenomes. These can be efficiently harvested to expand existing datasets without additional costs. Here, we present new mitochondrial genomes for six marine angelfish species (F: Pomacanthidae), assembled and annotated from off‐target UCE reads. We provide the first comparative analysis of all mitochondrial genomes available for the Pomacanthidae. Results showed that the average length of pomacanthid mitogenomes is 16.8 kbp. Total GC and AT content varied between 44.5% and 46.3%, and 53.7% and 55.5%, respectively. The architecture of angelfish mitogenomes was comparable to that seen in other fish species with 13 protein‐coding genes (PCGs), 22 transfer RNA genes, two ribosomal RNA genes and the control region. All 13 PCGs evolved under purifying selection, highlighting a high level of selection pressure and gene expression to preserve genetic integrity. The ND6 and ATP8 genes had the highest ratio of non‐synonymous (dN) to synonymous (dS) substitutions, indicating a relaxation of purifying selection constraints. Finally, these newly assembled mitogenomes will allow further investigations of the population genetics, systematics and evolutionary biology of one of the most prominent reef fish family in the aquarium trade.

## Graphical abstract

This study describes newly assembled and annotated mitogenomes harvested from off‐target reads of ultraconserved element captured data for six pomacanthid species. Secondly, it investigates evolutionary patterns of protein‐coding genes across all mitogenomes available for the family on the NCBI Reference Sequence Database. Finally, we generated the most comprehensive phylogenetic tree for the family using complete reference mitogenomes.

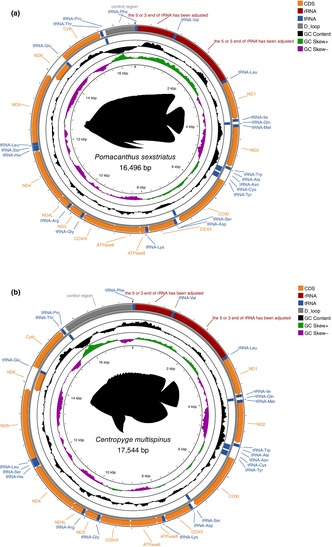

## 1 INTRODUCTION

Primarily designed to target specific regions of the genome, sequence capture (or enrichment) methods have the potential to recover complete mitochondrial genomes as a by‐product from off‐target sequences (do Amaral et al., 2015; Miller et al., 2022; Samuels et al., 2013; Smith et al., 2014). Leveraging these off‐target reads provides a valuable source of informative mitochondrial genes that can have broad applications ranging from species identification and environmental biomonitoring through DNA barcoding, population genetics or phylogenetic reconstructions (DeSalle & Goldstein, 2019; Drummond et al., 2015; Miller et al., 2022; Pereira, 2000; Zarza et al., 2016). Without the need for additional sequencing or mtDNA‐specific baits, DNA reference libraries can expand readily and rapidly at no further cost. This is particularly beneficial for non‐model or taxonomically underrepresented organisms where genetic coverage is limited. Retrospectively, this approach can also help enrich existing multilocus genetic datasets to maximise their utility and taxonomic or genetic sampling. In general, multi‐marker‐based datasets were shown to yield more accurate species identification, biodiversity assessment and phylogenetic relationships than single‐gene approaches (Baldauf et al., 2000; Hillis, 1996; Meyer & Paulay, 2005; Rubinoff & Holland, 2005). While the genomic era has provided the means for applying both reduced and whole genome methods in phylogenetic and evolutionary studies, there is still a place for comparing new datasets with published sequence data. In many cases, the extensive mitochondrial database available for species can be included in phylogenomic assessment through a holistic approach that involves extracting legacy markers from off‐target reads (do Amaral et al., 2015; Quattrini et al., 2023).

Increased sequencing of mitochondrial material improves our understanding of the composition, structure and variation of mitogenomes, subsequently providing a framework to investigate genetic processes underlying the evolution of lineages (Mercer et al., 2011; Satoh et al., 2016; Taanman, 1999). The fish mitochondrial genome is a highly conserved, circular and double‐stranded DNA molecule (Satoh et al., 2016) ranging from 15 to 24 kbp, as per NCBI GenBank Database in January 2024. It generally follows the same arrangement as other vertebrate mitogenomes, usually containing 37 genes of which 13 are protein‐coding genes (PCGs), two are ribosomal RNA (rRNA) genes, 22 are transfer RNA (tRNA) genes and one is a non‐coding control region (D‐loop) (Satoh et al., 2016). As the most speciose group of vertebrates, fishes, have been of particular interest to scientists aiming to unravel evolutionary mechanisms driving diversification processes (Brawand et al., 2014; Dornburg & Near, 2021; Friedman et al., 2022; Near et al., 2013; Volff, 2005). This includes the highly diverse reef fish lineages that inhabit coral reef systems worldwide.

The family Pomacanthidae contains some of the most iconic and recognisable reef fish species. Their striking colour patterns and dramatic change in coloration while transitioning from juvenile to adult stage made them among the most valued specimens in the ornamental fish trade today (Wabnitz, 2003). There are 90 valid nominal species and seven genera of marine angelfishes (Fricke et al., 2023). Complete mitochondrial genomes are available on the NCBI Reference Sequence database for only 33 pomacanthid species across the seven genera, representing 37% taxonomic coverage for the family. Several studies have examined the systematics and ancestral biogeography of pomacanthids using mitochondrial data (Alva‐Campbell et al., 2010; Baraf et al., 2019; DiBattista et al., 2012; Gaither et al., 2014) but no comprehensive comparative analysis of their mitogenomes has been conducted. In addition to expanding the NCBI Reference sequence database for the family Pomacanthidae, additional mitogenomic resources will allow further research aiming to delve into the genetic diversity, differentiation and evolutionary history of reef fishes. Moreover, marine angelfishes exhibit of the highest occurrence of hybridisation recorded among reef fishes (Tea et al., 2020), a biological process that can be challenging to uncover using nuclear markers which undergo recombination (Wallis et al., 2017). Past introgression might be more readily identifiable using mitochondrial DNA (mtDNA) rather than nuclear DNA due to its maternal inheritance and non‐recombining nature (Wallis et al., 2017). Although it will not be able to differentiate invasion‐driven from selection‐driven introgression (Seixas et al., 2018). Furthermore, asymmetric discordance between mitochondrial and nuclear markers can inform on degrees of sequence divergence and structural variation, helping to detect mitochondrial introgression (Toews & Brelsford, 2012; Wallis et al., 2017). Expanding the mitochondrial genome library for pomacanthids will serve as a valuable tool to help answer evolutionary questions about the family amidst the complexities introduced by hybridisation events.

**FIGURE 1:**
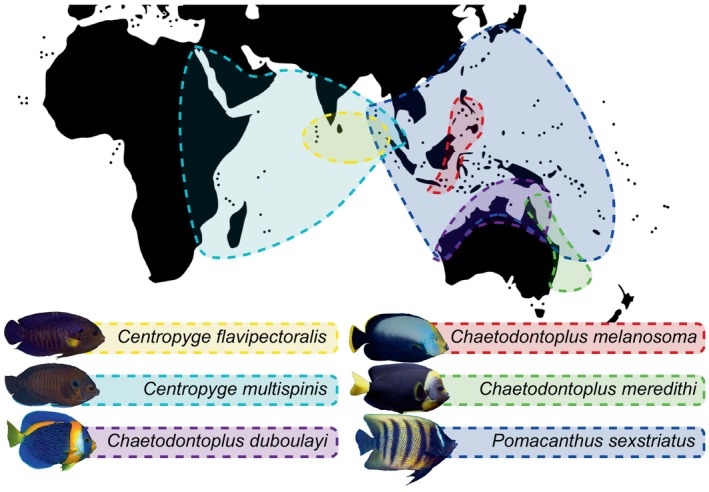
Geographical distribution of the six pomacanthid species sampled in this study. Pictures were provided by François Libert, Yi‐Kai Tea, Jake Adams or obtained from the Wikimedia Images Commons. GenBank accession numbers for the six assembled and annotated mitogenomes are as follow: *Centropyge flavipectoralis* [PP316127], *Centropyge multispinis* [PP316128], *Chaetodontoplus duboulayi* [PP316126], *Chaetodontoplus meredithi* [PP316124], *Chaetodontoplus melanosoma* [PP316125] and *Pomacanthus sexstriatus* [PP316129].

In the present study, we assembled and describe new mitogenomes for six pomacanthid species (Figure 1) before conducting comparative and phylogenetic analyses across all mitogenomes available on the NCBI Reference Sequence Database.

## 2 MATERIALS AND METHODS

### 2.1 Taxon sampling, UCE sequencing and data handling

Target capture of ultraconserved elements (UCEs) was conducted across 68 samples representing 45 distinct nominal species of Pomacanthidae or 50% of the family. We extracted DNA from tissue samples of 45 pomacanthid species using DNeasy Blood and tissue kits (Qiagen) following the manufacturer's instructions. Each sample was then quantified with a Qubit 2.0 Fluorometer using Qubit dsDNA BR Assay kits and Nanodrop spectrophotometer (Thermo Fisher Scientific Inc.) to measure DNA quality. Genomic DNAs were shipped to Arbor Bioscience (US) for library preparation and targeted enrichment following MyBaits v.5.0 protocol using the *acanthomorph* probe set (Alfaro et al., 2018). Adapters were trimmed off of raw reads in the illumiprocessor v.0.39 wrapper program (Faircloth et al., 2012) using the *trimmomatic* package (Bolger et al., 2014) with default parameters set for a minimum output sequence length of 40 bp and Phred quality score of 33. The targeted UCE dataset has been analysed in a separate study (Baraf et al., in preparation). Here we concentrate on harvesting mitogenomic data from the off‐target reads. To facilitate this, we built a reference mitogenomic database by downloading all 33 mitochondrial genomes for the family Pomacanthidae available on the NBCI Reference Sequence database. We manually verified that gene nomenclature was consistent across all genomes and standardised sequence's names when needed (e.g. control region changed to D‐loop).

### 2.2 Mitogenome assembly and annotation

Cleaned raw reads were assembled and scanned for mitochondrial contigs, and annotated using MitoFinder v.1.4.1 (Allio et al., 2020). Due to the fragmented nature of mitochondrial sequences found in capture data, we estimated coverage for all contigs following the mapping workflow described in https://phyluce.readthedocs.io/en/latest/daily‐use/daily‐use‐4‐workflows.html, and set a minimum contig size of 500 bp instead of the default 1000 bp. Using the MitoFinder workflow, mitochondrial contigs were assembled with MetaSPAdes assembler (Nurk et al., 2017) and annotated using BLAST v.2.12.0 against the reference mitogenomic database with a percentage of overlap in BLAST best hit set at 20% and a e‐value cut‐off ≤1e−06. Non‐standard animal mitochondrial genes and tRNA genes were annotated with the *‐‐new‐genes* option and MiTFi annotation pipeline (Juhling et al., 2012), respectively. A second round of annotation was conducted on the assembled mitochondrial contigs with the addition of the *‐‐adjust‐direction* option to establish the direction of contigs relative to those in the reference database. Final annotations were validated by assessing the consistency between annotations from MitoFinder and those from MitoAnnotator, a pipeline implemented in the Mitochondrial Genome Database of fish MitoFish v.3.90 (Iwasaki et al., 2013; Zhu et al., 2023). We manually inspected the contigs and their alignments to the reference mitogenomes in Geneious v.2023.2.1. Finally, we extracted all COI sequences obtained from MetaSPAdes and blasted them against the NCBI database using BLASTN v. 2.14.1 (Zhang et al., 2000) and Megablast (Morgulis et al., 2008) to confirm species identification.

### 2.3 Mitogenomes statistics

We calculated AT/GC‐skew to assess the nucleotide bias and analyse strand asymmetry to describe overall patterns of nucleotide composition following the equation AT‐skew = (A − T)/(A + T) and GC‐skew = (G − C)/(G + C) (Perna & Kocher, 1995). We used AliGROOVE v.1.08 (Kück et al., 2014) to examine nucleotide composition and divergence patterns of 13 PCGs for all six newly assembled and the 33 existing RefSeq pomacanthid mitogenomes. To tag potentially unreliable species relationships, we used the phylogenetic tree inferred from IQTree (Nguyen et al., 2015) as a guiding tree and calculated reliabilities of single branches. To assess degree of sequence heterogeneity, we also calculated site score for amino acid to compare it to nucleotide‐based results. To investigate evolutionary patterns in PCGs we calculated average non‐synonymous (dN) and synonymous (dS) substitutions values of all pairwise comparisons, and dN/dS ratios between alignments of 13 PCGs for all pomacanthid species in DnaSP v.5 (Librado & Rozas, 2009). Additionally, we estimated pairwise genetic distances from mt PCGs using Kimura's two‐parameter (K2P) model (Kimura, 1980) in MEGA v.11 (Tamura et al., 2021) and highest nucleotide diversity with R using the package *pegas*.

### 2.4 Alignment and phylogenetic analysis

Protein‐coding gene sequences for the 39 pomacanthid species and one outgroup species from the family Acanthuridae were aligned individually in Geneious using the MAFFT aligner (Katoh et al., 2002; Katoh & Standley, 2013) with the default parameters settings and manually curated when necessary. Multiple sequence alignments were then concatenated into a 75% complete matrix. After finding the best‐fit partition model by running ModelFinder combined with the greedy algorithm from PartitionFinder2, we inferred a maximum likelihood (ML) tree from the concatenated alignments in IQTree (Kalyaanamoorthy et al., 2017; Lanfear et al., 2017; Nguyen et al., 2015). Branch support was estimated by Ultrafast Bootstrap approximation (Hoang et al., 2017) and SH‐like approximate likelihood ratio test (Guindon et al., 2010) for 1000 bootstrap replicates.

## 3 RESULTS

### 3.1 Genome structure and nucleotide composition

**FIGURE 2:**
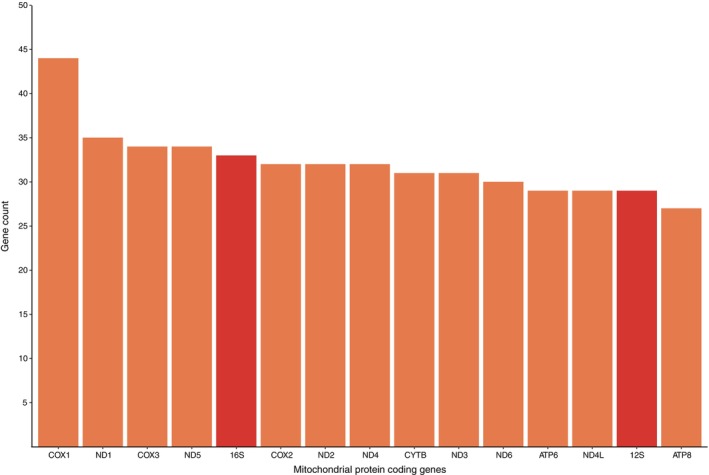
Total number of mitochondrial protein‐coding genes (orange) and rRNAs (red) retrieved from UCE reads after two MitoFinder runs on 68 samples across 45 pomacanthid species.

From the 68 samples representing 45 pomacanthid species, UCE reads yielded complete and partial mitogenomes for 12 (or 18% across samples) and 11 pomacanthid species, respectively. A total of 482 mitochondrial genes were found across all samples with the COI gene being the most frequently recovered marker (Figure 2). NCBI Megablast results on COI sequences had over 99% identity blast hits with conspecifics.

**FIGURE 3:**
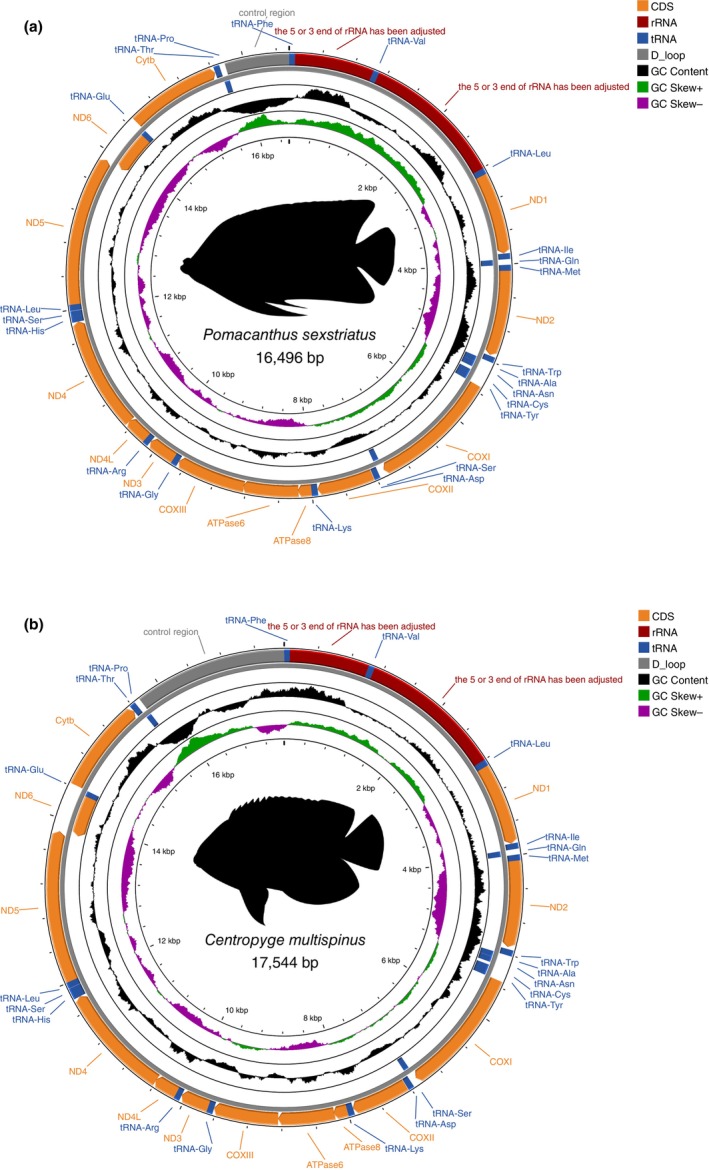
Mitochondrial genomes for two pomacanthid species, *Pomacanthus sexstriatus* and *Centropyge multispinis* of length 16,496 and 17,544 bp respectively. Circular gene maps were generated with cgview (Stothard & Wishart, 2005). Both mitogenomes contained 13 genes, two rRNAs and 22 tRNAs. Outer and inner circles represent the H‐strand and L‐strand of the mitochondrial DNA, respectively. Fish silhouette were drawn by L.M.B. Gene maps for the other four pomacanthid species are in Data S1.

**FIGURE 4:**
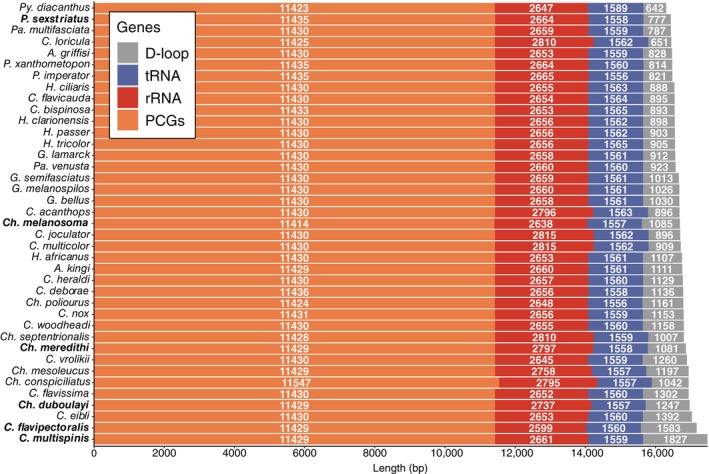
Summary of length of protein‐coding genes, tRNAs, rRNAs and D‐loop genes for six pomacanthid mitogenomes extracted from UCE data and 33 pomacanthid mitochondrial reference sequences obtained from NCBI GenBank. Abbreviations for genera are as follows: A, *Apolemichthys*; C, *Centropyge*; Ch, *Chaetodontoplus*; G, *Genicanthus*; H, *Holacanthus*; Pa, *Paracentropyge*; Po, *Pomacanthus* and Py, *Pygoplites*. Species names in bold are new assembled and annotated pomacanthid mitogenomes presented in this study. Accession numbers for mitogenomes obtained from NCBI GenBank are reported in Table S1.

Lengths of the six assembled pomacanthid mitogenomes ranged from 16,496 for *Pomacanthus sexstriatus* to 17,544 bp, for *Centropyge multispinis* (Figures 1, 3 and 4), with an average length of 17 kbp. Size difference across mitogenomes could be attributed in majority to the length variation of the control region (Figure 4). All mitogenomes (except for *Chaetodontoplus meredithi* and *Pomacanthus sexstriatus*) were assembled from complementary strands of the reference DNA sequences and had a reversed direction. This likely resulted from the short read lengths of off‐target sequences extracted from UCE captured data. Total GC and AT content varied from 44.6% to 46.3% and 53.7% to 55.5%, respectively, with a mean base composition of A: 27.7%, T: 26.6%, G: 16.5% and C: 29.1% (Table 1). The AT‐skews were all positive (0.005 to 0.035) to the exception of *C. flavipectoralis* whereas GC‐skews were all negative (−0.31 to −0.23, Table 1). All mitogenomes contained 37 genes – 13 PCGs (ND1, ND2, COXI, COXII, ATP8, ATP6, COXIII, ND3, ND4L, ND4, ND5, ND6 and CYTB), 22 tRNA genes, two rRNA (12S and 16S) genes and the control region (Figure 3). Mitochondrial ND6 and eight tRNA genes (Gln, Ala, Asn, Cys, Tyr, Ser, Glu and Pro) were encoded on the light strand (L‐strand) and the remaining 28 genes were encoded on the heavy strand (H‐strand). The control region was positioned between tRNA‐Pro and tRNA‐Phe, and had the highest length variation of all mitochondrial genes, ranging from 777 to 1827 bp (Figure 4).

**TABLE 1:** Summary of nucleotide composition of whole mitochondrial genomes for six pomacanthid species.

Species name	Size (bp)	GC (%)	AT (%)	Nucleotide composition				AT skew	GC skew
				A	T	G	C		
*Centropyge flavipectoralis*	17,251	45.4	54.3	27.1	27.5	17.4	28.0	−0.007326	−0.241685
*Centropyge multispinis*	17,544	46.1	53.9	27.1	26.8	17.3	28.7	0.00556586	−0.2478261
*Chaetodontoplus duboulayi*	17,021	46.3	53.7	27.6	26.1	15.9	30.4	0.02793296	−0.3131749
*Chaetodontoplus melanosoma*	16,771	45.1	55.1	28.2	26.8	16.1	28.9	0.02545454	−0.28444444
*Chaetodontoplus meredithi*	16,917	46.3	53.7	27.8	25.9	16.3	30.0	0.03538175	−0.2958963
*Pomacanthus sexstriatus*	16,496	44.6	55.4	28.7	26.8	15.9	28.7	0.03423423	−0.2869955

*Note*: Additional summary on gene features in Table S2. Strand asymmetry was calculated with the following formulas: AT skew = [(A − T)/(A + T)] and GC skew = [(G − C)/(G + C)] (Perna & Kocher, 1995).

Intergenic spacers (IGS) were found between ND1 and tRNA‐Ile (3–4 bp), ND2 and tRNA‐Trp (8–44 bp), tRNA‐Ala and tRNA‐Asn (1–2 bp), tRNA‐Asn and tRNA‐Cys (34–42 bp), tRNA‐Tyr and COX1 (1 bp), COX1 and tRNA‐Asp (6–8 bp), tRNA‐Lys and ATP8 (1–2 bp), tRNA‐Ser and tRNA‐Leu (3–5 bp), tRNA‐Glu and CytB (3–4 bp), D‐loop and tRNA‐Phe (1 bp). Lengths were generally consistent across species, with IGS between ND2 and tRNA‐Trp genes located on the H‐strand showing the most length variation (e.g. *Centropyge* species, Table S2). The largest IGS measured 42 bp and marked the separation between tRNA‐Asn and tRNA‐Cys (Table S2). We found overlap (Table S2) between the adjacent PCGs ATP8‐ATP6 (10 bp), ND4L‐ND4 (7 bp), ND5‐ND6 (4 bp) and ATP6‐COX3 (1 bp, Table S2).

### 3.2 Protein‐coding genes

**FIGURE 5:**
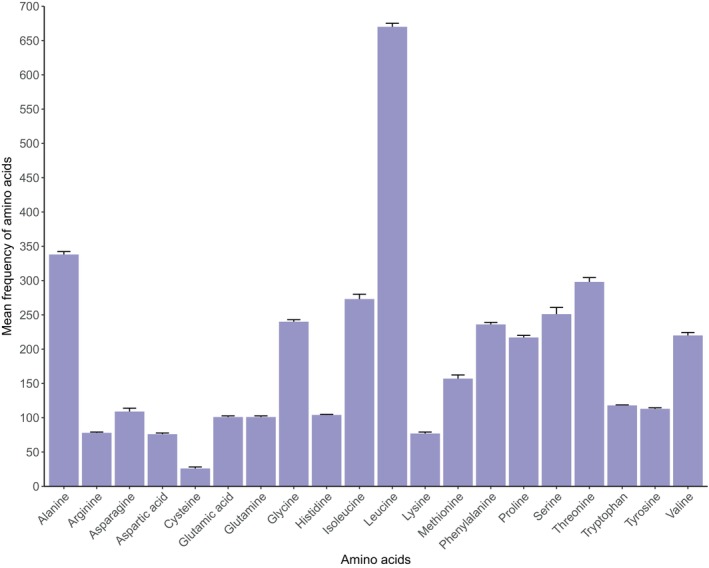
Mean frequency of amino acids in six pomacanthid mitogenomes. Error bars represent standard deviation. Details on codon usage can be found in the Table S3.

Combined length for the 13PCGs ranged from 11,414 to 11,430 bp across the six pomacanthid mitogenomes, representing around 68% of the mitogenome's total length (Figure 4). Twelve PCGs were encoded on the H‐strand while only ND6 was encoded on the L‐strand in reversed sequence (Figure 3). Two initiation codons for the amino acid methionine were identified (ATG/GTG) in the H‐strand PCGs (Table S2), with ATG being the most common across all six mitogenomes. ND6 had different initiation codons that coded for the amino acid Leucine (TTA/CTA). Incomplete termination codons (T*/TA*) were detected for PCGs COX1, ND3, ND4, CytB, COX3 and ATP6 (Table S2). Pomacanthid mitogenomes included between 3738 and 3800 codons (excluding stop codons). Across all amino acids, Leucine (669 ± 7.0) and Alanine (337 ± 3.5) had the highest frequency (Figure 5) with codons CTA/CTC/CTT and GCA/GCC found in highest proportions, respectively. Cysteine was the least predominant amino acid (26 ± 2.2). Overall, codon usage and amino acids were consistent across all pomacanthid species (Figure 5, Figure S2, Table S3).

**FIGURE 6:**
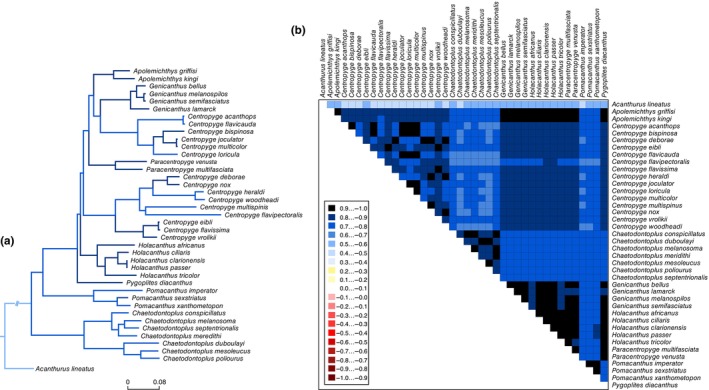
Sequence similarity score (a) guide tree and (b) matrix based on nucleotide composition of 13 PCGs for 39 complete mitochondrial genomes. The obtained mean similarity score (MSS) between sequences is represented by a coloured square. The site scores are ranging from −1, indicating low mean similarity score in sequence composition (red colouring) to +1, indicating higher mean similarity score with other sequence composition (blue colouring). Coloured branches represent branch reliability and are based on the mean score between corresponding terminal taxon and all other taxa. Internal scores are calculated by the mean of the total pairwise similarity scores between taxa which are connected by a branch.

**FIGURE 7:**
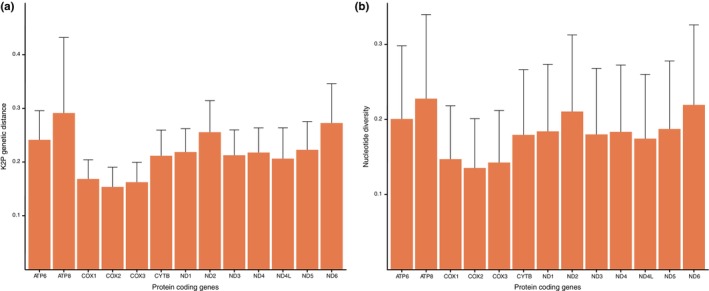
(a) Average nucleotide pairwise distances estimated in MEGA v.11 using Kimura 2‐parameter model and (b) Average nucleotide diversity π calculated in R using the package *pegas*, for 13 protein‐coding genes from 39 pomacanthid mitogenomes. Error bars represent standard deviations.

There was no heterogeneity in sequence divergence across PCGs of the 39 pomacanthid species with all sites displaying positive mean similarity scores (Figure 6). The paraphyletic *Centropyge* genus had the highest degree of sequence heterogeneity across species with an averaged MSS of 0.81 (±0.4). Heterogeneity was higher in the nucleotide dataset compared to amino‐acid dataset (Figure S3), reflecting variation in codon usage but not in amino acid production. Ratios ω of non‐synonymous (dN) to synonymous (dS) substitutions in the pomacanthid genomes revealed that all 13 PCGs evolved under purifying selection (ω < 1, Table 2) and had a high degree of similarity (ω < 0.30, Table 2). ND6 genes (ω = 0.27 ± 0.004) and ATP8 (ω = 0.18 ± 0.005) had the highest mutation rates while the COX1 gene had the lowest (ω = 0.01 ± 0.0002) across all mitogenomes (Table 2). Comparably to observed patterns of dN/dS, ATP8 and ND6 genes had the largest K2P distance and highest nucleotide diversity among the 39 pomacanthid mitogenomes followed by ND2 and ATP6 (Table 2, Figure 7). Interspecies K2P distances ranged from 0.153% (SD ± 0.037) for the COX2 gene to 0.291% (SD ± 0.141) for the ATP8 gene, which also showed the most variation in between species (Figure 7a). Nucleotide diversity ranged from 0.132 in the COX2 gene to 0.227 in the ATP8 gene but was consistent across species (Figure 7b).

**TABLE 2:** Summary of average non‐synonymous (dN) and synonymous (dS) substitutions values of all pairwise comparisons, and dN/dS ratios ω between alignments of 13 protein‐coding genes for 39 pomacanthid species.

PCGs	Length (bp)	dN	dS	dN/dS (±SD)
ATP6	681	0.06394	1.20781	0.053 (±0.029)
ATP8	168	0.17800	0.98076	0.181 (±0.15)
CYTB	1134	0.04435	1.25660	0.035 (±0.022)
COX1	1539	0.01178	1.06791	0.011 (±0.007)
COX2	690	0.01788	0.93425	0.019 (±0.013)
COX3	783	0.02171	0.90028	0.024 (±0.0005)
ND1	975	0.04155	1.22241	0.034 (±0.17)
ND2	1044	0.08364	1.11179	0.075 (±0.038)
ND3	348	0.04419	1.18896	0.037 (±0.021)
ND4	1380	0.04696	1.33760	0.041 (±0.021)
ND4L	297	0.03586	1.16750	0.031 (±0.026)
ND5	1836	0.06301	1.14503	0.055 (±0.030)
ND6	521	0.17242	0.62733	0.275 (±0.12)

### 3.3 Transfer and ribosomal RNA genes, and control region

Across all mitogenomes, the 22tRNAs had lengths ranging from 65 to 77 bp (Table S2), adding up to total lengths varying between 1557 and 1560 bp for *Ch. duboulayi* and *melanosoma*, and *C. flavipectoralis* respectively. All tRNAs were encoded on the H‐strand. Small and large rRNA genes were also located on the H‐strand, ranging from 949 to 958 bp and 1650 to 1844 bp respectively. They bordered the tRNA gene for Valine, as seen in other fish mitogenomes (Satoh et al., 2016). AT content for small and large rRNA genes varied between 50.7% and 55.2% [50.8/54.6 (*C. flavipectoralis*), 50.7/53.9 (*C. multispinis*), 51.9/53.8 (*Ch. duboulayi*), 51.5/55.0 (*Ch. melanosoma*), 51.3/54.8 (*Ch. meredithi*) and 51.2/55.2 (*P. sexstriatus*)]. The Control region or d‐loop region had the most variation in length across all mitochondrial genes, ranging from 447 to 1827 bp (Figure 2) and was located between tRNA‐Thr and tRNA‐Pro genes (Figure 3).

### 3.4 Phylogenetic analyses

**FIGURE 8:**
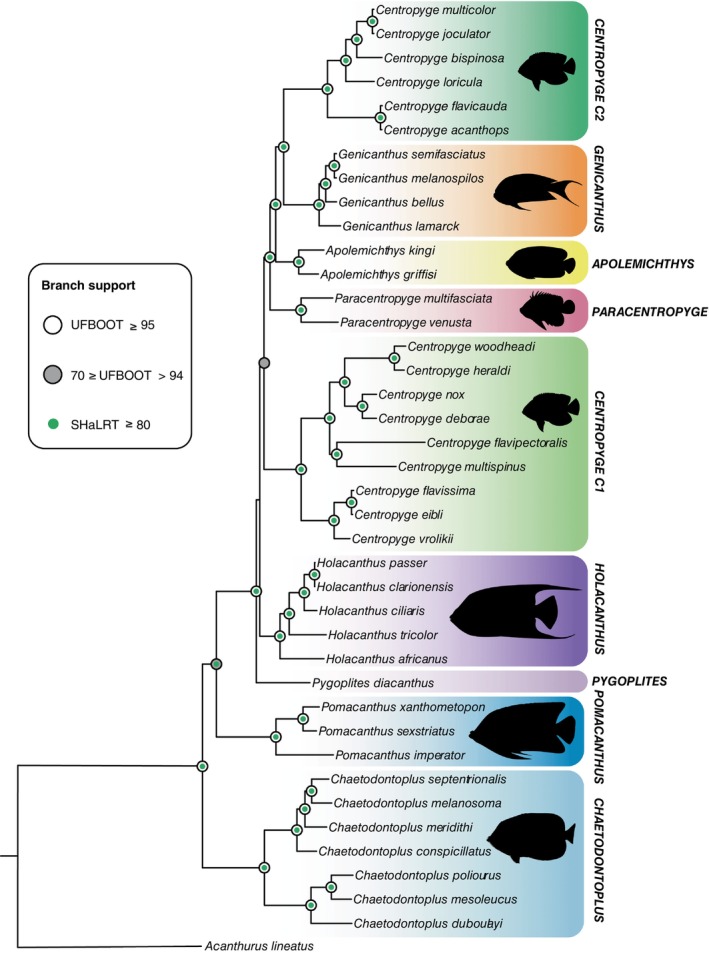
Inferred phylogeny of the Pomacanthidae generated from maximum likelihood (ML) analyses of 13 PCGs and two RNAs extracted from 39 mitochondrial genomes. Presented values correspond to SH‐like approximate likelihood ratio test (SH‐aLRT) and Ultrafast bootstrap (UFBoot2) analyses. Branch support values were selected following the IQ‐TREE Manual (Nguyen et al., 2015).

Final concatenated alignment comprised of 14,045 bp. The topology obtained for the 39 pomacanthid delineated nine strongly supported clades (Figure 8) with the exception of *Centropyge* clade 1 (C1, SH‐aLRT = 93.5, UFBOOT = 53), *Holacanthus* (SH‐aLRT = 53.3, UFBOOT = 39) and *Pomacanthus*. *Chaetodontoplus* was the first clade to diverge and received strong support (SH‐aLRT = 100, UFBOOT = 100). It was followed by the *Pomacanthus* lineage which had moderate support (SH‐aLRT = 80.4, UFBOOT = 84) and monotypic *Pygoplites* (SH‐aLRT = 100, UFBOOT = 100). The subsequent clade to diverge led to the *Holacanthus* genus, which had low support (SH‐aLRT = 53.3, UFBOOT = 39). Comparably, the position of *Centropyge C1* on the backbone of the pomacanthid tree was not well supported (SH‐aLRT = 93.5, UFBOOT = 53). However, the separation between the two commonly identified sub‐clades, delimiting species belonging to the *flavissima* ‘complex’ (C1.1) from the rest of the *Centropyge* species found within C1 (C1.2), was strongly supported (SH‐aLRT = 100, UFBOOT = 100). Following the branching out of C1, *Paracentropyge*, *Apolemichthys*, *Genicanthus* and *Centropyge C2* clades, were strongly supported (SH‐aLRT = 100, UFBOOT = 100, Figure 8).

## 4 DISCUSSION

Among the 13 complete pomacanthid mitogenomes obtained from off‐target regions in UCE sequencing data, six were not on the NCBI Reference Sequence Database, bringing the taxonomic coverage for the family to 43%. All assembled mitogenomes had similar composition and arrangement as commonly found in other vertebrate and fish mitogenomes (Pereira, 2000; Satoh et al., 2016) and measured around 16.8 kbp on average. Mitochondrial genes followed a distribution between L and H‐strands as previously observed in fish mitogenomes (Ruan et al., 2020) whereby ND6 and eight tRNA genes were encoded on the former while the remaining 28 genes were encoded on the H‐strand. The control region was located between tRNA‐Thr and tRNA‐Pro genes as seen in other fishes and had higher AT content, from 57.5% to 69%, than the rest of the mitochondrial genes. GC and AT content were similar across mitogenomes, approximately 45.6% and 54.4% respectively (Table 1). GC skew was above −0.23 for all mitogenomes (Table 1), indicating an increase in cytosine (C) content over guanine (G) within the nucleotide composition. Lengths of IGS and overlap in adjacent gene sequences were consistent with those observed for other reef fish families (Gao et al., 2023; Tang et al., 2023; Wang et al., 2022). For all six pomacanthid mitogenomes, it contained a conserved sequence motif (5′‐GCCGG‐3′) which was previously identified across some vertebrate species (Satoh et al., 2016), suggesting that this IGS can likely be linked to signals of initiation for L‐strand replication (O_L_; Broughton et al., 2001; Clayton, 1991; Satoh et al., 2016). The origin of H‐strand replication (O_H_) is expected to be within the control region or D‐loop gene (Clayton, 1991). As for many vertebrates, including fish, some incomplete termination codons might be completed by post‐transcriptional polyadenylation occurring during mRNA maturation (Gissi et al., 2008; Li et al., 2021; Nagaike et al., 2008; Ruan et al., 2020).

### 4.1 Comparative analysis

The 33 mitogenomes obtained from NCBI Reference Sequence database and the six newly assembled mitogenomes presented in this study were highly conserved in terms of their genome size, gene content and sequence divergence (Figures 4, 5, 6). As observed in other fish mitogenomes (Satoh et al., 2016), the control region showed the highest length variation of all mitochondrial genes, ranging from 777 bp for *P. sexstriatus* to 1827 bp for *C. multispinis* (Figure 4). All 13 PCGs evolved under negative or purifying selection (Table 2), which eliminates arising deleterious mutations, a pattern observed in other reef fish mitogenomes (Gao et al., 2023; Tang et al., 2023). This highlighted high level of selection pressure and gene expression to preserve the genetic integrity of pomacanthid mitogenomes. Mitochondrial genes ND6 and ATP8 had the highest ratio ω of non‐synonymous (dN) to synonymous (dS) substitutions (Table 2). This indicated that those genes were the most rapidly evolving PCGs in pomacanthid mitochondrial genomes. A relaxation of purifying selection constraints for those genes is a trend that has been observed in mitogenomes of sedentary fishes, which do not have as high energy demands as seen in more active fishes (Strohm et al., 2015). For instance, ND6 gene is involved in the production of the enzymatic protein NADH dehydrogenase 6, which plays an essential role in the initiation phase of the electron transport chain process. ATP8 protein participate in the functioning of the mitochondrial proton pump. Both genes are involved in the process of oxidative phosphorylation, which is the primary source of ATP for metabolic processes providing an important energy source for biosynthesis.

### 4.2 Phylogenetic analysis

The present analysis of 13 PCGs from 39 pomacanthid mitogenomes provides the most comprehensive assessment for the family Pomacanthidae using mitochondrial data to date. It was highly congruent with phylogenies previously inferred for the family based on limited and specific mtDNA and nuclear markers (Baraf et al., 2019; Frédérich et al., 2017) as well as the UCE‐based phylogeny from which these off read mitochondrial resources originate from (Baraf et al., 2024), though these results might vary with future increases in taxon sampling. Our latest phylogeny strongly supported the position of *Chaetodontoplus* at the root of the tree (Figure 8), however this clade has sometimes been recovered as sister to *Pomacanthus* (Baraf et al., 2019; Frédérich et al., 2017), which only received moderate support from phylogenetic analyses. The present branching arrangement, whereby *Chaetodontoplus* is first to diverged followed by *Pomacanthus*, agrees with UCE‐based phylogeny for the family (Baraf et al., 2024). Phylogenetic discordance for the divergence of these two pomacanthid lineages might result from ancient introgression involving extinct or outgroup lineages at the base of the pomacanthid tree (Baraf et al., in preparation). The most recent common ancestor of *Centropyge* C1 and *Holacanthus* received low support from phylogenetic analyses (Figure 8). The relationship between *Pygoplites* and *Holacanthus* genera relative to each other and the backbone of the pomacanthid tree has been challenging to resolve. Some phylogenetic reconstructions have one or the other lineage diverging first or forming a sister group relationship (Baraf et al., 2019; Frédérich et al., 2017), however none of these topological arrangements received strong support suggesting that underlying biological or evolutionary factors might be obscuring the recovery of a clear phylogenetic signal. Whole mitochondrial genomes yielded similar results whereby *Pygoplites* initially diverged and received strong support whereas the subsequent origins of the *Holacanthus lineage* was poorly supported. Strong incomplete lineage sorting for *Pygoplites* and *Centropyge* C1 has been identified as an important factor impacting the recovery of inter‐generic relationships in phylogenetic reconstructions (Baraf et al., 2024). The closeness between these nodes and the *Holacanthus* branch (1 or 2 partitions) might in part explain for the low support of the clade in several phylogenies for the family. Note that the mitogenome available on the NCBI Reference Sequence Database for *Centropyge interrupta* (NC_026451.1) appeared to be misidentified as the species was recovered as part of the *Genicanthus* genus in phylogenetic analyses. A BLAST search of the COI sequence from this mitogenome reference confirmed this misidentification and consequently, it was removed from our reference database.

## 5 CONCLUSIONS

Pomacanthid mitogenome measured 16.8 kbp on average and was highly conserved across species. All six mitogenomes assembled and presented in this study comprised of 37 genes, including 13 PCGs, 22 tRNA genes, two rRNA genes and one control region. It followed a genetic structure that has been observed in other vertebrates and fish mitogenomes. AT‐skews were mostly all positives while GC‐skews were all negatives. The most frequent start codon and amino acid were ATG and Leucine respectively. We found no heterogeneity in sequence divergence across pomacanthid species. While all PCGs were under purifying selection, ND6 and ATP8 genes showed some degree of relaxed purification and higher mutation rates, which might be reflecting the sedentary lifestyle of pomacanthid species. Moreover, the ATP8 gene showed the most genetic variation across the family. Downstream examination of mitogenomes will provide new insights into interspecific genetic variation, gene function and diversification processes of marine angelfish.

## AUTHOR CONTRIBUTIONS

**Lauriane M. Baraf:** Conceptualization (lead); data curation (lead); formal analysis (lead); funding acquisition (supporting); investigation (lead); methodology (lead); visualization (lead); writing – original draft (lead). **Julia Y. Hung:** Resources (lead); writing – review and editing (supporting). **Morgan S. Pratchett:** Supervision (supporting); validation (equal); writing – review and editing (supporting). **Peter F. Cowman:** Conceptualization (supporting); funding acquisition (lead); supervision (lead); validation (lead); writing – review and editing (lead).

## CONFLICT OF INTEREST STATEMENT

The authors declare no conflicts of interest.

## Supporting information


Data S1:

## Data Availability

Complete mitochondrial genomes assembled and annotated in the present study are available on the NCBI GenBank Database. Accession numbers are as follow: *Chaetodontoplus meredithi* [PP316124], *Chaetodontoplus melanosoma* [PP316125], *Chaetodontoplus duboulayi* [PP316126], *Centropyge flavipectoralis* [PP316127], *Centropyge multispinis* [PP316128] and *Pomacanthus sexstriatus* [PP316129]. Alignments and tree files are available on FigShare at https://figshare.com/s/675722dd87d5be753ab5 and bioinformatics scripts on GitHub https://github.com/Lavarchus/Mitogenomes.git.
