# Supplementary material for: Comparative mitogenomics of marine angelfishes (F: Pomacanthidae)
Source: Ecol Evol. 2024 Aug 8;14(8):e70127. doi: 10.1002/ece3.70127 (PMC11307104; doi:10.1002/ece3.70127)
Supplement: Supplementary file 1 — Data S1: [file ECE3-14-e70127-s001.zip › Supplementary Material_revised.docx]

**Supplementary Material.**

**Table S1.** Summary of all mitogenomes for pomacanthid species available on the NCBI Reference Sequence. Data were obtained in June 2023 and are sorted alphabetically by species' scientific names.

| Species common name | Scientific name | Length (in bp) | Accession number | Authors |
| --- | --- | --- | --- | --- |
| Armitage angelfish  (hybrid *Apolemichthys trimaculatus* X *Apolemichthys xanthurus)* | *Apolemichthys armitagei* | 16,551 | NC_027857 | Shen et al. (2015) |
| Griffis angelfish | *Apolemichthys griffisi* | 16,528 | NC_027592 | Shen et al. (2015) |
| Tiger angelfish | *Apolemichthys kingi* | 16,816 | NC_026520 | Shen et al. (2015) |
| Orangeback or Flameback angelfish | *Centropyge acanthops* | 16,748 | NC_028717 | Shen et al. (2015) |
| Coral beauty | *Centropyge bispinosa* | 16,772 | NC_028287 | Renshaw, M.A., Olds, B.P., Li, Y., Pfrender, M.E. and Lodge, D.M. |
| Blue velvet angelfish | *Centropyge deborae* | 16,843 | NC_036676 | Shen, K.N. and Hsiao, C.-D. |
| Blacktail angelfish | *Centropyge eibli* | 17,101 | NC_027971 | Shen et al. (2015) |
| Orange angelfish | *Centropyge flavicauda*  *(syn. C. fisheri)* | 16,747 | NC_028716 | Shen et al. (2015) |
| Lemonpeel angelfish | *Centropyge flavissima* | 17,010 | NC_027970 | Shen et al. (2015) |
| Yellow angelfish | *Centropyge heraldi* | 16,836 | NC_027968 | Shen et al. (2015) |
| Japanese angelfish | *Centropyge interrupta* | 16,151 | NC_026451 | Shen et al. (2015) |
| Yellowhead angelfish | *Centropyge joculator* | 16,778 | NC_027652 | Shen et al. (2015) |
| Flame angelfish | *Centropyge loricula* | 16,509 | NC_009872 | Yamanoue et al. (2007) |
| Pearlback angelfish | *Centropyge multicolor* | 16,791 | NC_027651 | Shen et al. (2015) |
| Midnight angelfish | *Centropyge nox* | 16,871 | NC_034953 | Shen et al. (2015) |
| Purplemask angelfish | *Centropyge venusta* | 16,641 | NC_027598 | Shen et al. (2015) |
| Pearlscale angelfish | *Centropyge vrolikii* | 16,966 | NC_036949 | Fernandez-Silva et al. (2018) |
| Woodhead’s angelfish | *Centropyge woodheadi* | 16,863 | NC_027969 | Shen et al. (2015) |
| Conspicuous angelfish | *Chaetodontoplus conspicillatus* | 16,988 | NC_026883 | Shen et al. (2015) |
| Vermiculated angelfish | *Chaetodontoplus mesoleucus* | 16,998 | NC_026521 | Hsiao et al. (2015) |
| Greytail angelfish | *Chaetodontoplus poliourus* | 16,961 | NC_044485 | Shen, K.N. |
| Bluestriped angelfish | *Chaetodontoplus septentrionalis* | 16,833 | NC_009873 | Yamanoue et al. (2007) |
| Ornate angelfish | *Genicanthus bellus* | 16,734 | NC_027597 | Hsiao et al. (2015) |
| BlackStriped angelfish | *Genicanthus lamarck* | 16,616 | NC_027972 | Hsiao et al. (2015) |
| Spotbreast angelfish | *Genicanthus melanospilos* | 16,736 | NC_027967 | Hsiao et al. (2015) |
| Japanese swallow angelfish | *Genicanthus semifasciatus* | 16,722 | NC_027596 | Hsiao et al. (2015) |
| Guinean angelfish | *Holacanthus africanus* | 16,819 | NC_027585 | Shen et al. (2015) |
| Queen angelfish | *Holacanthus ciliaris* | 16,606 | NC_027595 | Shen et al. (2015) |
| Clarion angelfish | *Holacanthus clarionensis* | 16,615 | NC_026453 | Shen et al. (2015) |
| King angelfish | *Holacanthus passer* | 16,620 | NC_027594 | Shen et al. (2015) |
| Rock beauty | *Holacanthus tricolor* | 16,625 | NC_027586 | Shen et al. (2015) |
| Barred angelfish | *Paracentropyge multifasciata* | 16,502 | NC_027599 | Shen et al. (2015) |
| Emperor angelfish | *Pomacanthus imperator* | 16,538 | NC_026304 | Ye et al. (2015) |
| Yellowface angelfish | *Pomacanthus xanthometopon* | 16,533 | NC_026303 | Shen et al. (2015) |
| Regal angelfish | *Pygoplites diacanthus* | 16,313 | NC_026545 | Shen et al. (2015) |

a)


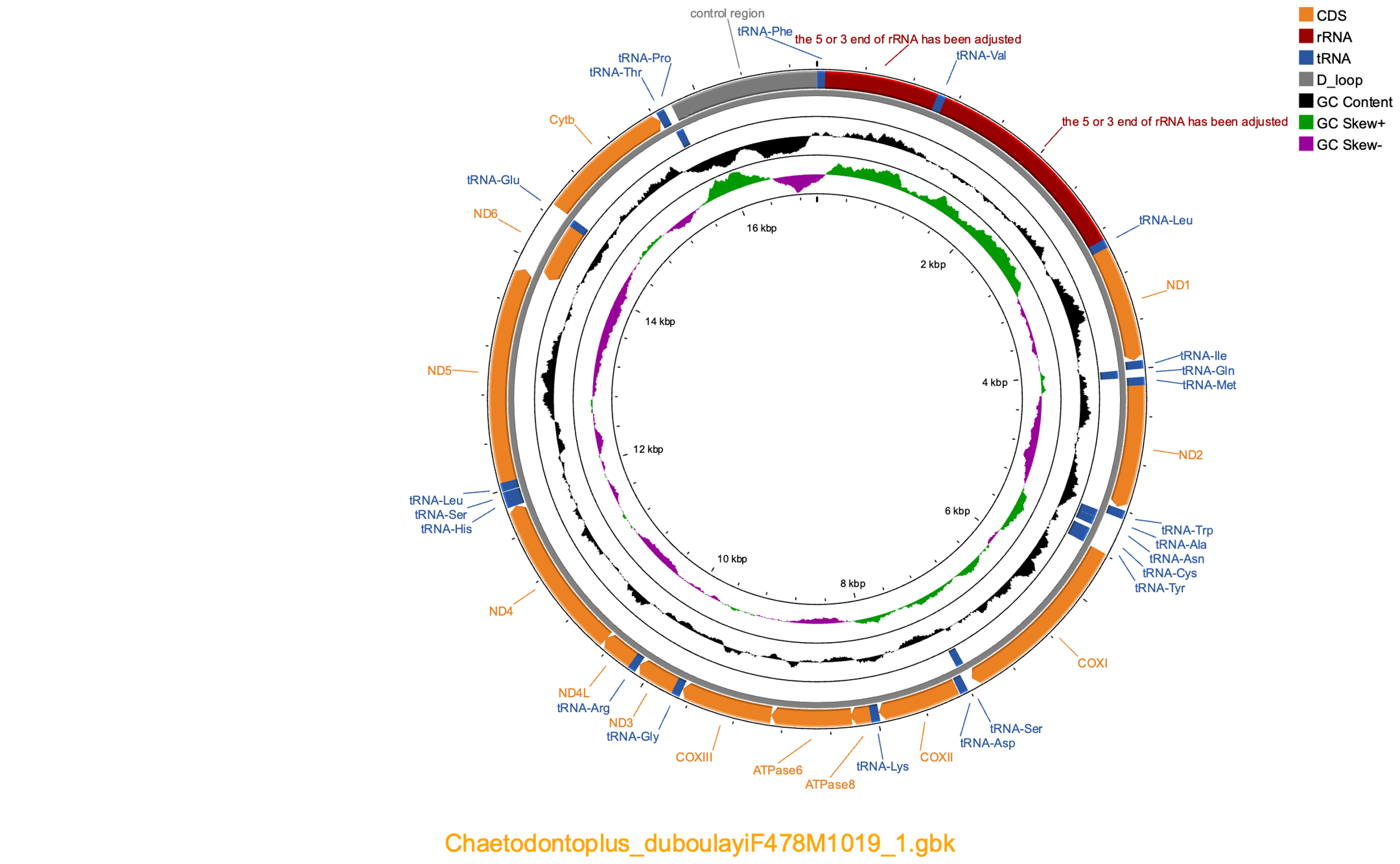


b)


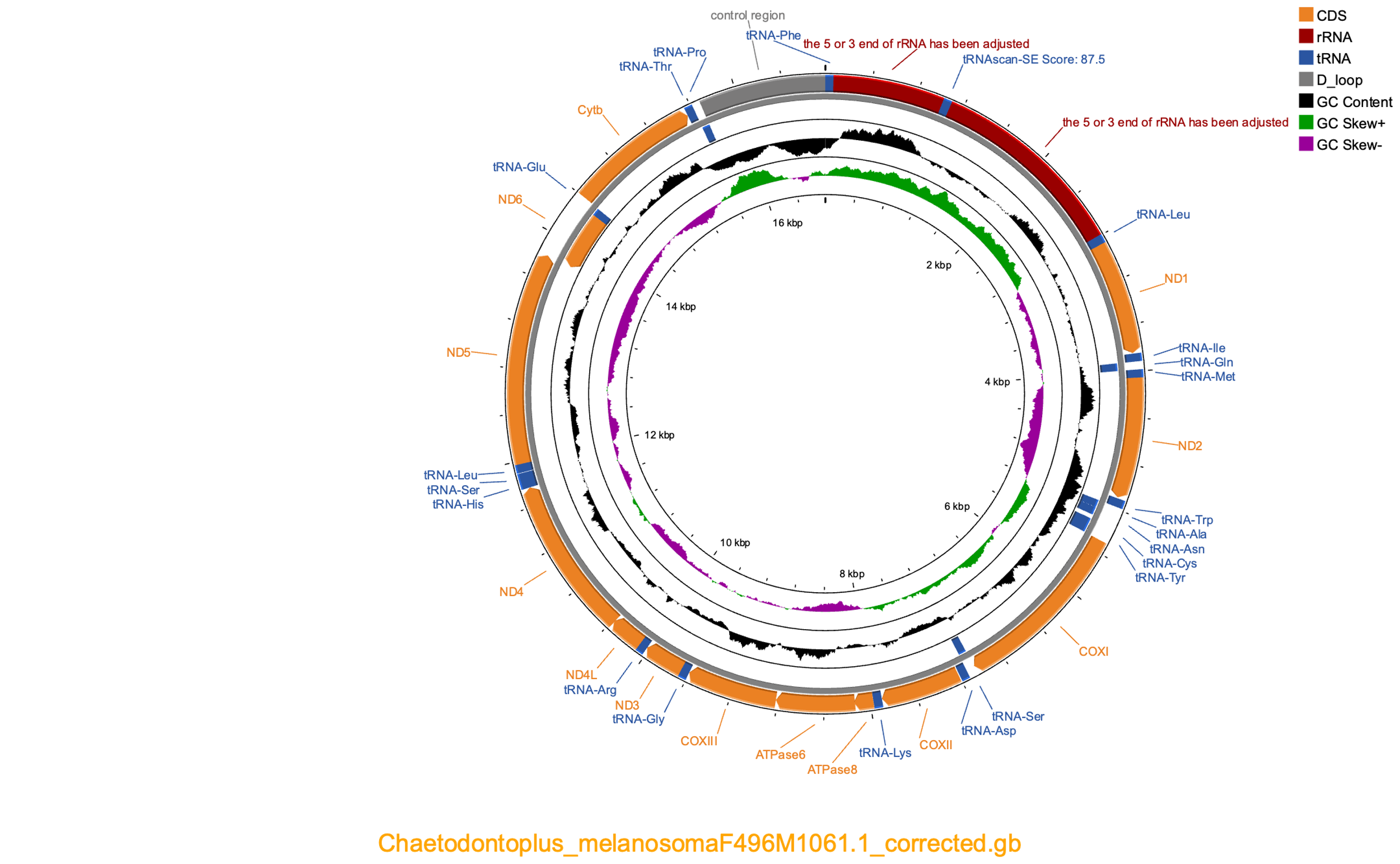


c)


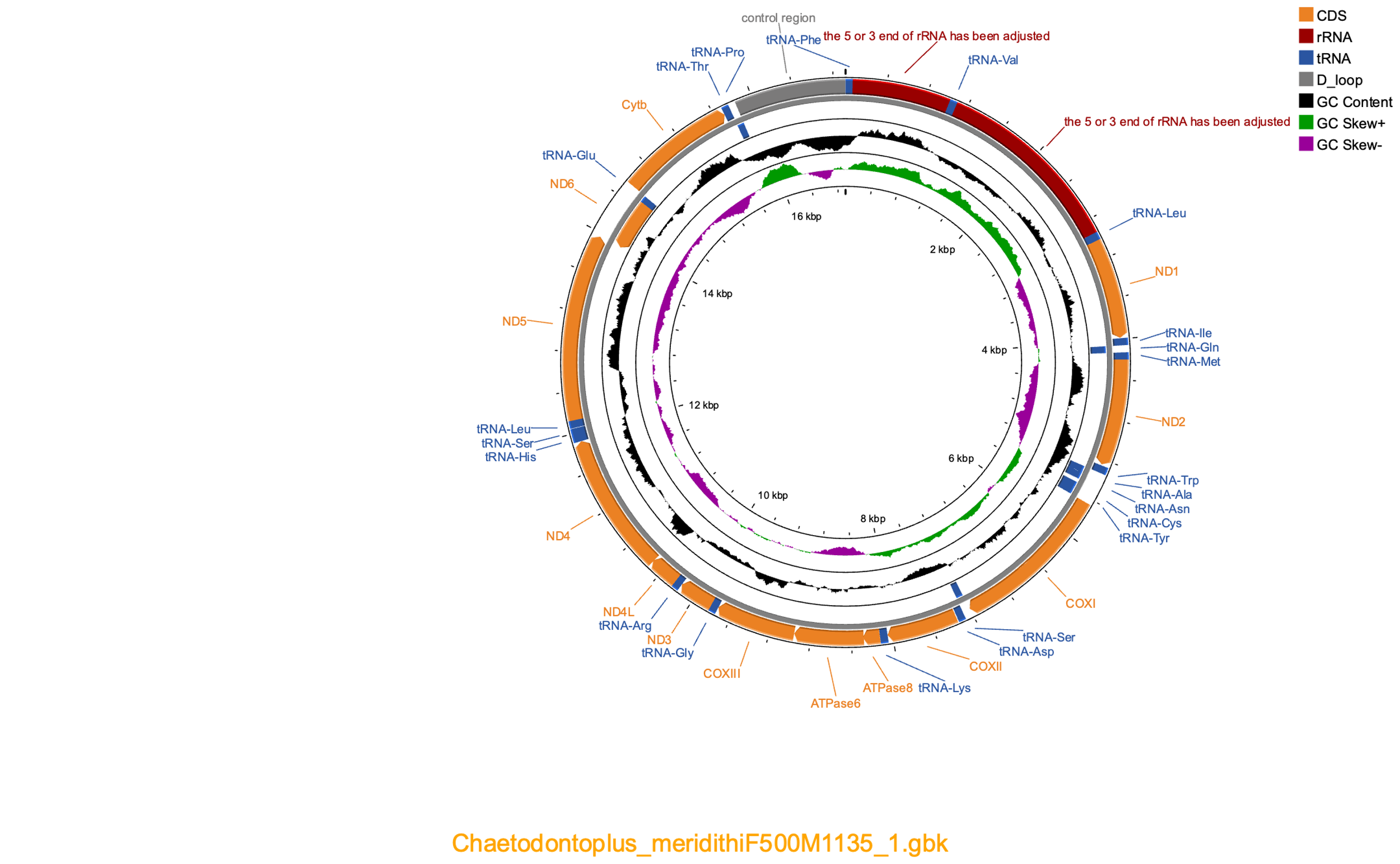


d)


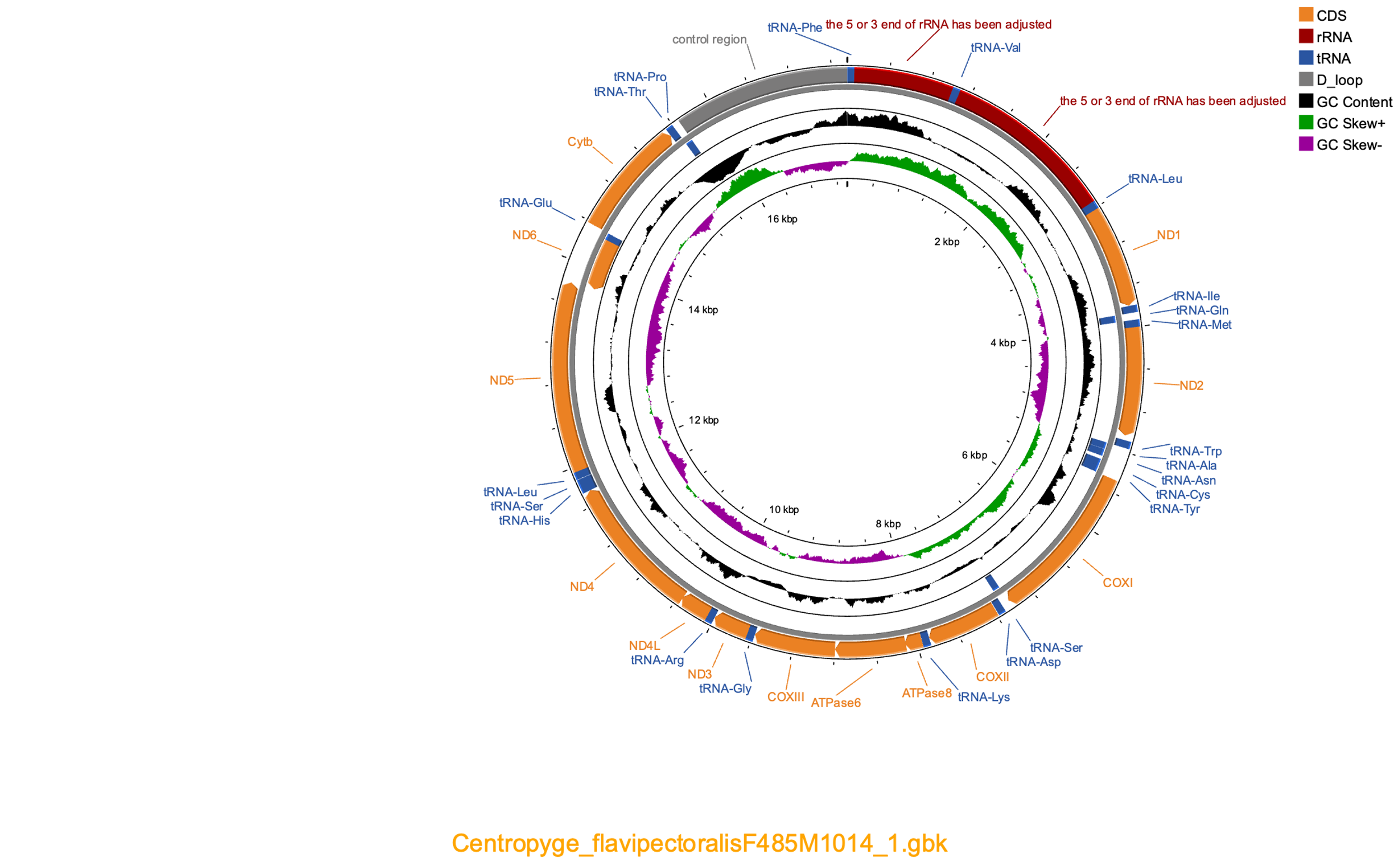


**Figure S1.** Circular gene maps for four pomacanthid species (not shown in main manuscript) a) *Chaetodontoplus duboulayi*, b) *Chaetodontoplus melanosoma*, c) *Chaetodontoplus meredithi,* d) *Centropyge flavipectoralis*, generated with cgview. All mitogenomes contained 13 genes, 2 rRNAs and 22 tRNAs. Outer and inner circles represent the H-strand and L-strand of the mitochondrial DNA, respectively.

**Figure S2.** Number of amino acids across six pomacanthid species. Numbers on bars represent values above 100. Amino acids not shown were not present in the dataset.

**Table S3.** Summary of codon usage across six pomacanthid species. Sp 1-6 are *C. flavipectoralis*, *C. multispinis*, *Ch. duboulayi*, *Ch. melanosoma*, *Ch. meredithi* and *P. sexstriatus*, respectively. Amino acid (AA) abbreviations are as follows: Alanine (A), Cysteine (C), Aspartic acid (D), Glutamic acid (E), Phenylalanine (F), Glycine (G), Histidine (H), Isoleucine (I), Lysine (K), Leucine (L), Methionine (M), Asparagine (N), Proline (P), Glutamine (Q), Arginine (R), Serine (S), Threonine (T), Valine (V), Tryptophan (W), Tyrosine (Y).

|  | | AA (%) | | | | | |
| --- | --- | --- | --- | --- | --- | --- | --- |
| Codon | **AA** | ***sp1*** | ***sp2*** | **sp3** | **sp4** | **sp5** | **sp6** |
| GCA | A | 33.6 | 32.4 | 29.5 | 33.9 | 29.1 | 31.5 |
| GCC | A | 37.1 | 40.0 | 40.7 | 41.7 | 43.6 | 42.0 |
| GCG | A | 8.5 | 6.8 | 4.8 | 3.3 | 5.9 | 4.7 |
| GCT | A | 20.8 | 20.9 | 25.0 | 21.0 | 21.4 | 21.9 |
| TGC | C | 72.0 | 66.7 | 79.2 | 75.0 | 70.8 | 80.0 |
| TGT | C | 28.0 | 33.3 | 20.8 | 25.0 | 29.2 | 20.0 |
| GAC | D | 72.0 | 71.1 | 77.6 | 74.4 | 74.4 | 63.0 |
| GAT | D | 28.0 | 28.9 | 22.4 | 25.6 | 25.6 | 37.0 |
| GAA | E | 70.7 | 73.0 | 79.8 | 79.2 | 77.7 | 76.2 |
| GAG | E | 29.3 | 27.0 | 20.2 | 20.8 | 22.3 | 23.8 |
| TTC | F | 44.7 | 40.9 | 62.2 | 50.9 | 62.0 | 54.4 |
| TTT | F | 55.3 | 59.1 | 37.8 | 49.1 | 38.0 | 45.6 |
| GGA | G | 25.3 | 30.1 | 33.6 | 35.3 | 31.3 | 33.9 |
| GGC | G | 35.9 | 36.4 | 39.1 | 36.5 | 42.1 | 40.6 |
| GGG | G | 24.1 | 22.6 | 14.5 | 12.4 | 15.8 | 13.4 |
| GGT | G | 14.7 | 10.9 | 12.8 | 15.8 | 10.8 | 12.1 |
| CAC | H | 67.9 | 76.9 | 78.8 | 69.5 | 73.1 | 63.1 |
| CAT | H | 32.1 | 23.1 | 21.2 | 30.5 | 26.9 | 36.9 |
| ATC | I | 36.9 | 43.6 | 55.3 | 40.8 | 53.0 | 41.4 |
| ATT | I | 63.1 | 56.4 | 44.7 | 59.2 | 47.0 | 58.6 |
| AAA | K | 76.0 | 70.3 | 90.8 | 88.8 | 81.0 | 88.2 |
| AAG | K | 24.0 | 29.7 | 9.2 | 11.3 | 19.0 | 11.8 |
| CTA | L | 23.1 | 24.2 | 21.6 | 28.4 | 25.1 | 26.3 |
| CTC | L | 20.9 | 23.2 | 31.1 | 23.5 | 27.7 | 24 |
| CTG | L | 9.0 | 8.3 | 6.8 | 6.0 | 7.2 | 5.9 |
| CTT | L | 24.6 | 25.7 | 22.4 | 24.1 | 21.8 | 23.1 |
| TTA | L | 17.8 | 14.3 | 14.4 | 15.5 | 15.0 | 16.8 |
| TTG | L | 4.7 | 4.4 | 3.8 | 2.5 | 3.2 | 3.9 |
| ATA | M | 64.6 | 65.3 | 63.8 | 70.0 | 71.2 | 67.9 |
| ATG | M | 34.8 | 33.3 | 35.5 | 29.4 | 28.2 | 31.4 |
| GTG | M | 0.6 | 1.4 | 0.7 | 0.6 | 0.6 | 0.6 |
| AAC | N | 64.0 | 74.5 | 71.7 | 61.5 | 70.3 | 63.5 |
| AAT | N | 36.0 | 25.5 | 28.3 | 38.5 | 29.7 | 36.5 |
| CCA | P | 21.8 | 18.8 | 19.3 | 23.1 | 19.9 | 20.6 |
| CCC | P | 45.8 | 50.2 | 54.6 | 52.5 | 49.3 | 45.8 |
| CCG | P | 5.6 | 7.0 | 6.0 | 2.7 | 1.4 | 4.7 |
| CCT | P | 26.9 | 23.9 | 20.2 | 21.7 | 29.4 | 29.0 |
| CAA | Q | 81.4 | 80.6 | 86.1 | 89.9 | 77 | 87.8 |
| CAG | Q | 18.6 | 19.4 | 13.9 | 10.1 | 23 | 12.2 |
| CGA | R | 48.7 | 64.9 | 53.2 | 55 | 55.7 | 59.7 |
| CGC | R | 19.2 | 20.8 | 24.7 | 27.5 | 26.6 | 18.2 |
| CGG | R | 17.9 | 6.5 | 11.7 | 6.3 | 8.9 | 13.0 |
| CGT | R | 14.1 | 7.8 | 10.4 | 11.3 | 8.9 | 9.1 |
| AGC | S | 15.1 | 18.8 | 18.5 | 17.1 | 18.0 | 17.8 |
| AGT | S | 8.8 | 5.5 | 2.7 | 3.5 | 3.1 | 5.1 |
| TCA | S | 24.8 | 21.1 | 20.0 | 23.6 | 20.7 | 24.6 |
| TCC | S | 21.0 | 27.7 | 36.5 | 34.1 | 35.2 | 31.8 |
| TCG | S | 6.7 | 4.7 | 3.5 | 3.9 | 6.3 | 3.4 |
| TCT | S | 23.5 | 22.3 | 18.8 | 17.8 | 16.8 | 17.4 |
| ACA | T | 28.5 | 33.6 | 33.8 | 33 | 33.8 | 37.9 |
| ACC | T | 43.1 | 38.5 | 39.9 | 40.9 | 42.2 | 43.0 |
| ACG | T | 7.5 | 4.6 | 4.1 | 5.2 | 3.7 | 3.6 |
| ACT | T | 21.0 | 23.4 | 22.2 | 21.0 | 20.3 | 15.5 |
| GTA | V | 34.2 | 29.0 | 30.0 | 27.8 | 26.0 | 37.4 |
| GTC | V | 28.4 | 29.9 | 30.4 | 29.1 | 35.4 | 30.3 |
| GTG | V | 9.9 | 11.8 | 8.8 | 10.8 | 9.9 | 8.5 |
| GTT | V | 27.5 | 29.4 | 30.9 | 32.3 | 28.7 | 23.7 |
| TGA | W | 73.1 | 76.9 | 83.1 | 88.1 | 89 | 88.9 |
| TGG | W | 26.9 | 23.1 | 16.9 | 11.9 | 11 | 11.1 |
| TAC | Y | 47 | 59.3 | 64.3 | 55.8 | 64 | 56.5 |
| TAT | Y | 53 | 40.7 | 35.7 | 44.2 | 36 | 43.5 |

**Figure S3.** AliGROOVE similarity score analysis based on amino acid composition of 13 PCGs for 34 pomacanthid mitochondrial genomes.
